# A participatory community case study of periurban coastal flood vulnerability in southern Ecuador

**DOI:** 10.1371/journal.pone.0224171

**Published:** 2019-10-25

**Authors:** Erica Tauzer, Mercy J Borbor-Cordova, Jhoyzett Mendoza, Telmo De La Cuadra, Jorge Cunalata, Anna M Stewart-Ibarra

**Affiliations:** 1 Institute for Global Health & Translational Science, SUNY Upstate Medical University, Syracuse, New York, United States of America; 2 Facultad de Ingeniería Marítima y Ciencias del Mar, Escuela Superior Politecnica del Litoral (ESPOL), Guayaquil, Guayas Province, Ecuador; 3 National Service for Risk Management and Emergencies, Guayaquil, Guayas Province, Ecuador; 4 Universidad Tecnica de Machala, Machala, El Oro Province, Ecuador; 5 Department of Medicine, SUNY Upstate Medical University, Syracuse, New York, United States of America; 6 InterAmerican Institute for Global Change Research (IAI), Montevideo, Department of Montevideo, Uruguay; Johns Hopkins University, UNITED STATES

## Abstract

**Background:**

Populations in coastal cities are exposed to increasing risk of flooding, resulting in rising damages to health and assets. Adaptation measures, such as early warning systems for floods (EWSFs), have the potential to reduce the risk and impact of flood events when tailored to reflect the local social-ecological context and needs. Community perceptions and experiences play a critical role in risk management, since perceptions influence people’s behaviors in response to EWSFs and other interventions.

**Methods:**

We investigated community perceptions and responses in flood-prone periurban areas in the coastal city of Machala, Ecuador. Focus groups (n = 11) were held with community members (n = 65 people) to assess perceptions of flood exposure, sensitivity, adaptive capacity, and current alert systems. Discussions were audio recorded, transcribed, and coded by topic. Participatory maps were field validated, georeferenced, and digitized using GIS software. Qualitative data were triangulated with historical government information on rainfall, flood events, population demographics, and disease outbreaks.

**Results:**

Flooding was associated with seasonal rainfall, El Niño events, high ocean tides, blocked drainage areas, overflowing canals, collapsed sewer systems, and low local elevation. Participatory maps revealed spatial heterogeneity in perceived flood risk across the community. Ten areas of special concern were mapped, including places with strong currents during floods, low elevation areas with schools and homes, and other places that accumulate stagnant water. Sensitive populations included children, the elderly, physically handicapped people, low-income families, and recent migrants. Flood impacts included damages to property and infrastructure, power outages, and the economic cost of rebuilding/repairs. Health impacts included outbreaks of infectious diseases, skin infections, snakebite, and injury/drowning. Adaptive capacity was weakest during the preparation and recovery stages of flooding. Participants perceived that their capacity to take action was limited by a lack of social organization, political engagement, and financial capital. People perceived that flood forecasts were too general, and instead relied on alerts via social media.

**Conclusions:**

This study highlights the challenges and opportunities for climate change adaptation in coastal cities. Areas of special concern provide clear local policy targets. The participatory approach presented here (1) provides important context to shape local policy and interventions in Ecuador, complimenting data gathered through standard flood reports, (2) provides a voice for marginalized communities and a mechanism to raise local awareness, and (3) provides a research framework that can be adapted to other resource-limited coastal communities at risk of flooding.

## Introduction

Damages caused by flooding are growing in urban areas [1,2] due to increased population and assets, a changing climate [3], coastal subsidence [4–6], and deforestation [7,8]. In Latin America and the Caribbean (LAC), 7.5 million inhabitants and $299 billion USD in built capital are exposed to flooding from a 100-year event, without considering hurricanes [9]. This exposure will increase to 8.8 to 9.9 million inhabitants by mid-century, when taking into account extreme sea levels, increasing populations, and the historical trend in storm activity [9]. Flooding presents a high social and economic burden, particularly in low-income vulnerable populations—those who are least able to cope with the impacts and recover from the damages of flood events.

To address flooding and other disasters, the global Sendai Framework for Disaster Risk Reduction (2015–2030) identifies “understanding disaster risk” as a top priority [10]. A better understanding of local community perceptions of flood hazards can inform risk management planning that aims to reduce exposure to flood events while strengthening the resilience and adaptive capacity of communities [10,11].This understanding can inform the development of tailored climate services for disaster managers, such as early warning systems for floods (EWSFs) [10,12,13] When implemented effectively as part of a comprehensive risk management plan, a well-designed EWSF increases community and ecosystem resilience, reduces vulnerability and reduces damages to economies, health, property, infrastructure, and other assets of people, communities, nations, and the private sector [12,14].

Coastal Ecuador is particularly vulnerable to flooding due to an extensive, densely populated coastline along the Pacific Ocean [9]. This region experiences severe floods during El Niño events due to increased local rainfall [15]. A recent study identified southern coastal Ecuador as the location with the highest coastal risk in LAC due to a combination of coastal hazards, geographic exposure, and socioeconomic vulnerability [16]. When flood costs are measured as a percentage of GDP, the coastal city of Guayaquil, Ecuador, (population 2,644,891, [17]) ranks as the 3^rd^ most vulnerable city to flooding worldwide [18]. In these coastal cities, unstructured rapid urbanization has pushed the poor into low-lying areas along estuarine waterways prone to flooding [19,20].

In Ecuador, the Secretary of Risk Management (SNGR) has the primary responsibility to establish early warning systems with a multi-hazard approach in collaboration with national technical-scientific institutions, civil defense, and local governments [21]. EWSFs have been implemented in most of the river basins throughout the country using basic data; however, detailed hydrometeorological data are available in four hydrographic basins only. While these data are a key part of the national EWSF, it is widely understood that limited economic resources have impacted the operational capacity of EWSFs (A. Vaca, *pers*. *comm*.). The SNGR are also responsible for strengthening capacities for disaster prevention and recovery at municipal and local levels [22]. However, we hypothesize that a lack of engagement with community stakeholders has, in part, limited the local adaptive capacity and the implementation of adaptation actions, such as EWSFs.

Here we present a case study of community perceptions and responses to coastal urban flooding in LAC. Community perceptions and experiences play a critical role in risk management, since perceptions influence people’s behaviors in response to interventions and policies. We hypothesize that (1) flood exposure is multifactorial—driven by hydroclimatic events and local geographies, (2) flood risk is spatially heterogenous at the sub-neighborhood level, (3) populations are differentially sensitive to flooding, (4) impacts of flooding present a high social and economic burden in periurban communities, and (5) the community’s capacity to take actions in response to flooding depends on social, political, and financial assets. This study emerged in response to research priorities identified by the SNGR, an active partner in this investigation. Findings from this study inform the design and implementation of flood risk reduction actions, such as EWSFs, and adaptive capacity strengthening in Ecuador and in other regions with similar characteristics.

## Methods

### Ethics statement

This study was conducted in collaboration with the local municipal government of Machala and the SNGR. The investigation protocol was reviewed and deemed exempt by the Institutional Review Board (IRB) of SUNY Upstate Medical University. The study was also approved by the SNGR. All participants were over the age of 18 and no personal identifying information was collected. Due to the conversational approach used with the focus groups and potential literacy limitations, verbal consent was most appropriate and written consent was not deemed necessary by the IRB committee. Verbal consent was recorded in audio recordings of focus group conversations.

### Study area

The midsized port city of Machala (population 279,887) is located on the southern coast of Ecuador, and is the capital of El Oro province [17]. In a recent analysis of urban coastal risks in LAC, El Oro was identified as the top risk hotspot due to the large at-risk population (mostly located in Machala), susceptible ecosystems, high rate of flood exposure from El Niño events, and high level of social vulnerability (i.e., high infant mortality, high malnutrition, low income and high inequality) [20]. The economy of Machala is based on agriculture (bananas, cacao, coffee), aquaculture (shrimp), mining, and commerce associated with a major port and proximity to the Peruvian border. The city was settled on lowland mangrove forests and has an estuarine inlet along the Gulf of Guayaquil [23]. Machala grew through a rapid unstructured process of mangrove deforestation for shrimp farms and urban settlements, resulting in modification of the local hydrology of the mangroves and flood-prone slums bordering the mangrove fragments at the urban periphery [24].

The tropical climate is marked by a hot rainy season from January to April (average maximum temp = 31.7° in April) during which 79% of total annual rainfall occurs. Heavy rainfall is associated with El Niño events [15,25,26], such as the exceptionally strong 1997–1998 event, when over 1800 mm of rainfall were recorded. El Niño events occur cyclically (every 2 to 7 years) when tropical central and eastern Pacific Ocean surface temperatures increase, resulting in local climate anomalies [27].

**Fig 1:**
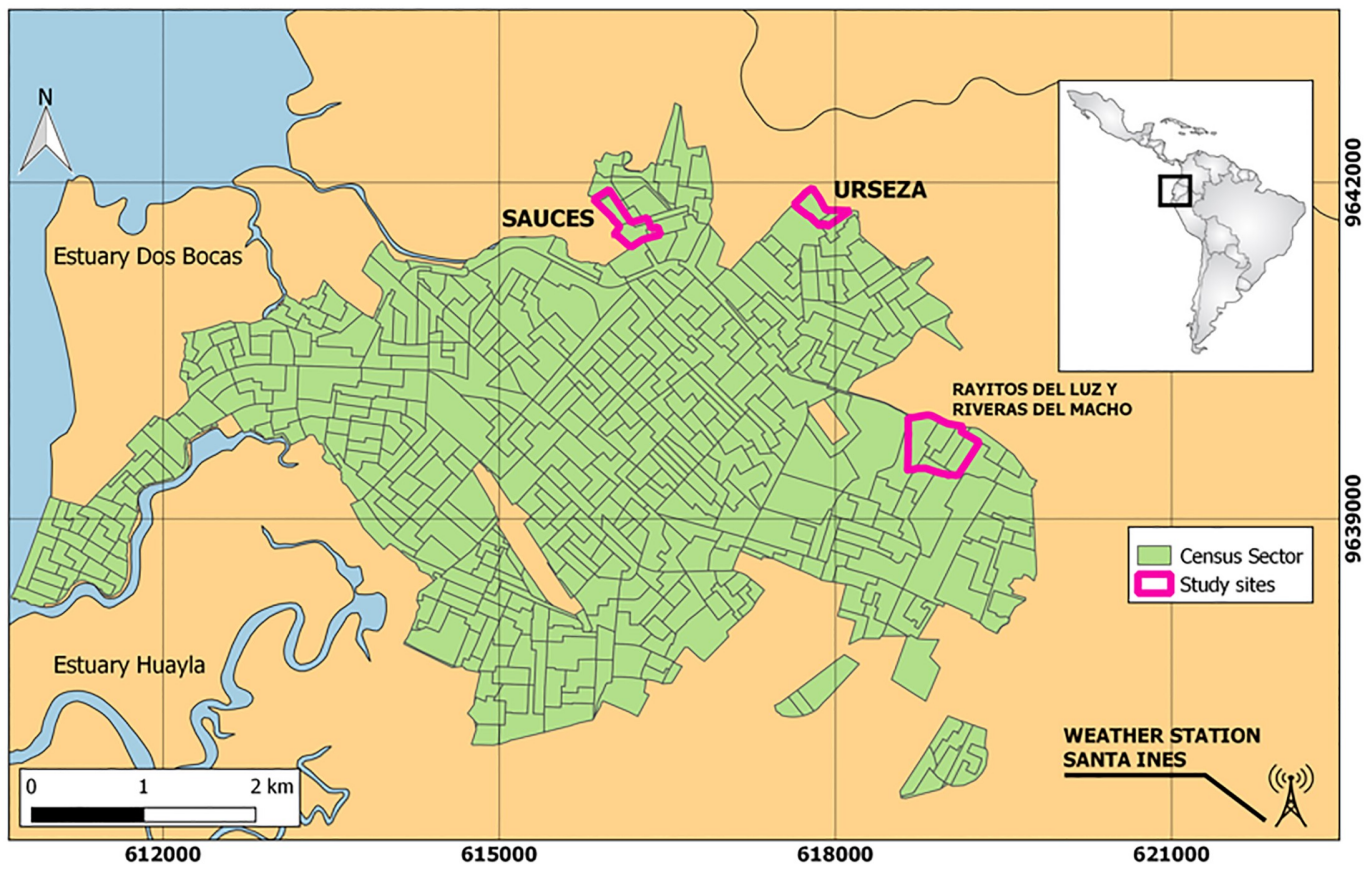
Urban study sites in the city of Machala, El Oro Province, Ecuador. The three participating study sites included areas of the cities noted by authorities as high flood risk areas. This map was created using freely available country boundary data from GADM.org and rendered in QGIS.

Three neighborhoods in the urban periphery were selected as study sites. These were identified as high flood risk zones through discussions with the municipal government and local SNGR officials (Fig 1). The sites were located 1.5–3 km apart. Site 1 was Sauces 2, a neighborhood adjacent to an abandoned shrimp farm (pop. 1266). Site 2 was Urseza 2 Sector 3, a neighborhood adjacent to a local river system (pop. 498). Site 3 included Rayito de Luz and Riveras del Macho, two neighborhoods adjacent to a large drainage canal (combined pop. 2,258). Neighborhood characteristics from the most recent national census are presented in Table 1. Generally, these communities lacked adequate access to urban infrastructure, such as paved streets, garbage collection, and municipal water/sewer connection.

**Table 1:** Demographic summary of study sites.

Census Indicator	Site 1: Sauces 2	Site 2: Urseza 2 Sector 3	Site 3: Riveras de Macho*	Site 3: Rayito de Luz*
2010 population	1,266	498	78	2,180
Maximum education of the head of household is primary education (% households)	57%	45%3	39%	51%
Age of the household (average years)	24.1	24.6	26.2	26.4
Households (%) with four or more people per bedroom	25%	18%	17%	16%
Women head of households (% households)	36%	31%	31%	31%
Households (%) without access to paved streets	54%	68%	43%	62%
Households (%) without access to sewerage	2%	0%	67%	66%
Households (%) without access to garbage collection	76%	63%	79%	83%
Households (%) without access to piped water inside the home	77%	77%	62%	70%

Demographic characteristics of study neighborhoods in Machala, Ecuador, from the most recent national census, conducted in 2010.

*Riveras de Macho and Rayito de Luz are treated as one study site, as they are geographically contiguous

### Research framework

**Fig 2:**
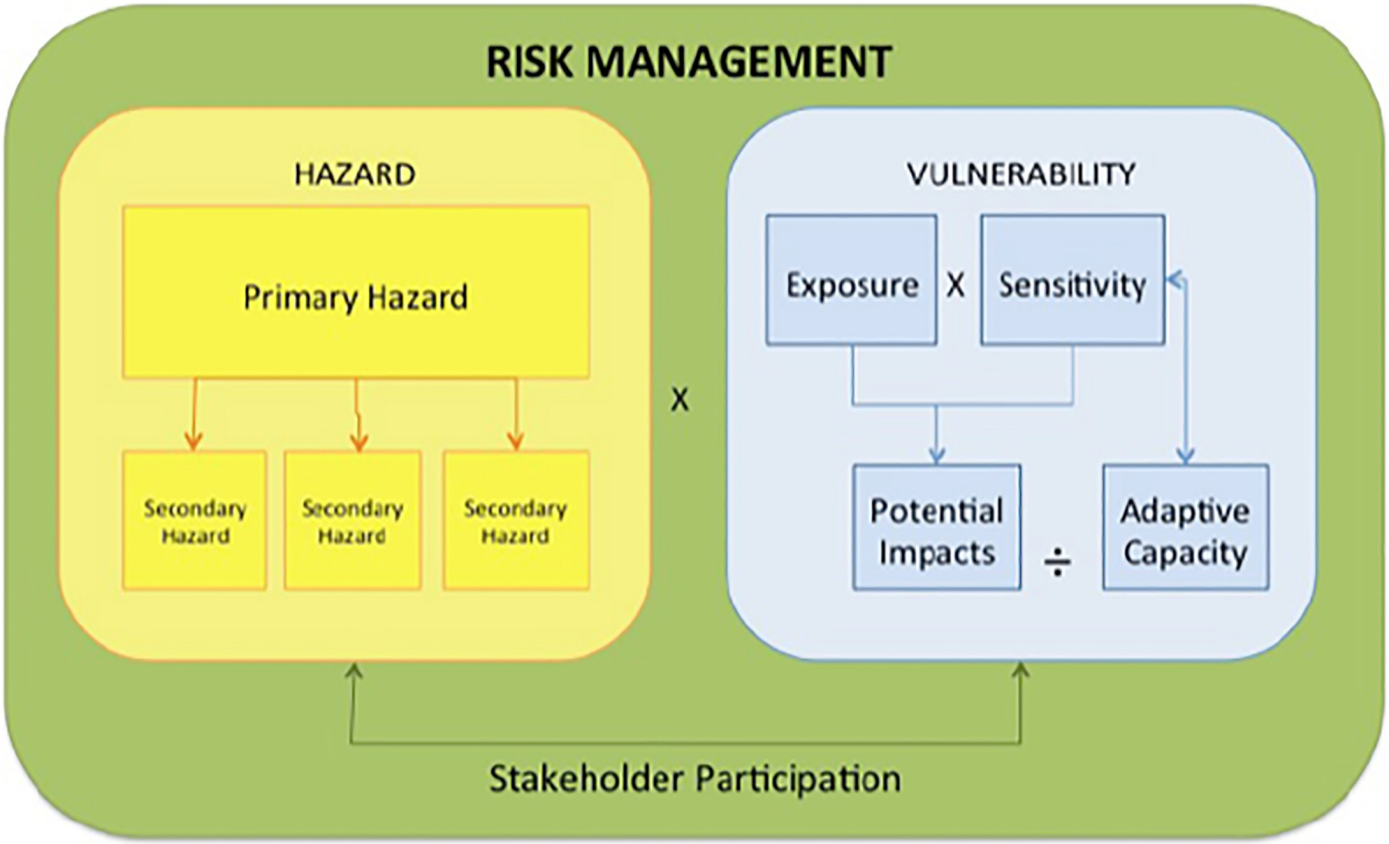
A research framework for flood hazards and vulnerability within the context of risk.

In this study, we utilize a research framework that is situated in the context of disaster risk reduction by encompassing the measures of hazard exposure, vulnerability, sensitivity, impact, and adaptive capacity [25] (Fig 2). Risk is described as a measure of the probability and severity of a given hazard and the consequences of those hazards on the normal functioning of the community [28–30]. Risk analysis is often highly quantitative, overlooking contextual social, cultural and historic dimensions [28,30–32]. In response, researchers have called for systems thinking (e.g. Haimes 2009 [28]) to overcome narrow risk definitions and the inclusion of “qualitative” normative risk characterization. The framework presented here is designed to bring localized, qualitative information into broader-scale risk or hazard analysis (e.g. the Global Natural Disaster Hotspot framework by Dilley et al., 2005 [33]). We draw on previously proposed and more generalized frameworks (i.e., [34–38]), and adapt these frameworks to include indicators more usable in a context of flooding in LAC, as done in prior studies of urban heat [39].

The primary metrics in this study were flood hazards and vulnerabilities (Fig 2). Primary hazards were defined as direct impacts from flooding events (e.g., high velocity flows destroying houses or eroding roads or bridges). Secondary hazards are those stemming from primary hazards, such as contaminated drinking water, power outages, or interruptions to communication or transportation systems. Flood vulnerability metrics include exposure, sensitivity and adaptive capacity [34,38]. Exposure to flooding refers to the presence of communities in places that could be adversely affected by flood hazards. Sensitivity, also called “susceptibility,” refers to the physical predisposition of exposed individuals or communities to be negatively affected by a flood event due to lack of resistance or predisposition to suffer harm as a consequence of a flood event [40–42]. Impacts (e.g., health, economic, social) result from an individual’s exposure combined with their unique sensitivities. Adaptive capacity refers to the ability of the system to respond or adjust to a flooding event, to moderate potential damage, to take advantage of opportunities, and to cope with the transformations that occur as a result of the flooding [40]. As a sub-category of adaptive capacity, this framework examines livelihood capitals [43,44]–the vital resource bases of communities and households (e.g., human, natural, financial, physical and social capitals).

### Focus group methodology

Semi-structured focus group discussions were held in each community. Neighborhood residents were recruited to participate in focus group discussion in consultation with the presidents of each neighborhood council, and when applicable (in two communities), the presidents of the councils of women. When possible, groups were segmented by gender to elicit a more open dialogue, based on our prior experience. Each focus group met twice. Both meetings were led by a moderator and documented by a group note taker who summarized the discussion on poster paper in real-time. The first focus group included questions regarding people’s previous experiences with flooding, specifically the causes and impacts of flooding, frequency of flooding, areas flooded, and the relative intensity of flood exposure. With facilitated guidance from the moderator, participants generated a timeline of severe flood events in each community; they self-defined these severe events as floods that exceeded their perception of normal (shallower) annual flooding. They noted the depth of normal flooding and specific severe flood events, and they listed the causes and impacts of flooding. A mapping exercise was conducted to identify high-risk areas and flood extent in each area during normal years and during moderate and extreme flood events. Focus group participants divided into groups of 3–5 people and were provided printed aerial imagery maps of their neighborhood. To orient themselves, participants first marked the location of important neighborhood landmarks (e.g. their homes, schools, soccer fields). Participants then outlined the areas impacted by the flooding events identified in the timeline, and they identified other areas of special concern with respect to flooding (e.g., areas with inadequate infrastructure). Map elements were verified by researchers and community leaders who walked through the community and georeferenced key landmarks and locations using handheld GPS units.

Within three weeks the same groups reconvened for a second focus group to discuss flood-related actions taken in their community, specifically preparation actions taken before flood events, response actions during flooding, and recovery actions post-flooding. People were also asked to identify key institutional partners associated with the actions, community assets, and resource limitations.

All focus group discussions were held in the late afternoon or evening in a community meeting area and lasted between 60 and 90 minutes. Representatives from the SNGR were present at every meeting to answer questions once the focus group was over. One to two researchers facilitated the focus groups and were accompanied by local research assistants who were trained as observers and note takers. All discussions were tape-recorded and transcribed with permission from participants. Local research assistants transcribed the recordings of focus groups.

We analyzed transcripts using codebook and qualitative analysis software, Dedoose (Version 6.1.11), a program that is commonly used to organize transcripts and documents (https://www.dedoose.com/) and to provide initial analysis of data. After creating a codebook, this software allowed us to tag pieces of text with the different codes. We then analyzed the text to identify when and where these codes appeared in the focus groups. Researchers later verified codes by assessing the context of the broader focus group conversation. The codebook was developed using the research framework described in Fig 2; codes are presented in S1 Table. Participant-generated histories of flood events (years and flood depth) were converted into bar charts for each site. Participant-generated maps of flood extent, areas of special concern, and community landmarks were digitized using Q-GIS and ArcGIS. Our findings were presented to communities and to the municipal government in January 2015 for feedback and validation.

### Government data sources

Focus group data were triangulated with available government data. Daily rainfall was provided by the National Institute for Meteorology and Hydrology (INAMHI) for the Granja Santa Ines weather station in Machala (1986–2015, 3°17’26” S, 79°54’5” W, 10 m above sea level). This was the closest station to the study sites, located approximately 5 km from the city center. We calculated total annual rainfall and number of days with heavy rainfall, defined as days with greater than 50 mm of rainfall—the 99^th^ percentile of daily rainfall during rainy months (baseline January to June, 1986–2015). All flood events in Machala were extracted from the publically available Desinventar database from 1990 to 2013 (n = 45 reports) [45] (https://online.desinventar.org/desinventar/#ECU-DISASTER). We reviewed the description of each event and coded the flood causes and impacts. Infectious disease outbreaks in Machala were identified in the Desinventar database [45] and from a prior analysis of dengue fever case data from the Ministry of Health [46]. We assessed available indicators of population sensitivity from the most recent national census (2010) [47], and compared focus group results to previously mapped neighborhood-level census data [48]. We also examined flood hazard maps for the city produced by the SNGR in 2015; hazard calculations by the SNGR were derived from elevation, slope and rainfall [49].

## Results

Eleven semi-structured focus group discussions, with a total of 65 people, were held from September 2014 to November 2014 (three groups in Site 1, four groups each in Sites 2 and 3). Groups in Sites 2 and 3 were segmented by gender, while in Site 1, a single group met comprised of both men and women based on local recommendations (but featured mostly women). A third meeting was held in Sauces 2 to obtain additional information on the evaluation of a recent flood prevention activity—the organization of a local effort to clear debris from drainage canals. Six to 25 people participated in each focus group, with ages ranging from late teens to late 70s.

### Exposure and flood hazards

**Fig 3:**
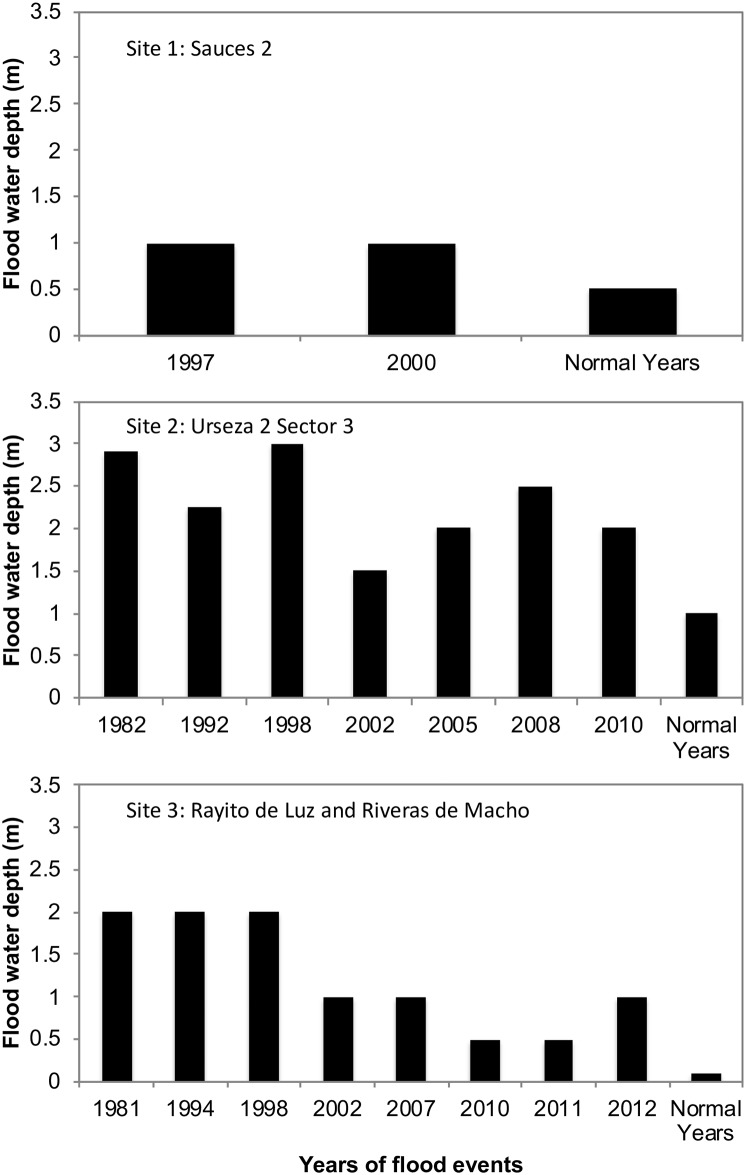
Historical flood timeline. A timeline of severe flood events and floodwater depth over the last 30 years was created by community members. The depth of floodwater in normal years is also noted.

Community members reported normal annual floods following heavy seasonal rains (floodwater depth range: 0.1 to 1 meter) (Fig 3). They identified multiple severe floods over the last 30 years (range: two events at Site 1 to eight events at site 3). Floodwater depths in severe floods ranged from 0.5 to 3 meters, and floods lasted hours to months. The most severe floods occurred in 1982 and 1997/98, which coincided with strong El Niño events. When compared to rainfall data, at least one of the three sites reported severe flooding in all years with high rainfall; bolded years in Table 2 show years that exceeded the upper quartile (740 mm/year) of total annual rainfall from 1990–2013). However, they also noted serious flooding in low rainfall years such as Site 3 in 1994 and Site 1 in 2000, and Site 2 in 2005. Community-reported flooding coincided with flood events reported at the city level in the Desinventar database, except at Site 1 in 2000. Not surprisingly, heavy rainfall was identified a cause of flooding in 14 of 17 years with flooding in government reports.

**Table 2:** Community-reported severe flood events compared to official reports of rainfall, flood causes, impacts and disease outbreaks.

Year	Sites with severe flooding	Flood events (n)	Annual rainfall (mm)	Days > 50 mm rainfall*	Causes^1^	Impacts^2^	Disease outbreaks^3^
1990		0	162	0			D [45]
1991		0	450	2			C [45]
**1992**	**Site 2**	**4**	**1089**	**4**	**R, EN, HT**	**T, C, H, HH**	**C & M**[45]
1993		2	693	1	R	T, C, H	T [45], M [50]
1994	Site 3	1	348	0	HT	E	
1995		2	482	3	R	C	
1996		0	351	0			C [45]
**1997**	**Site 1**	**7**	**1276**	**7**	**R, EN OF, HT**	**C, I, S, E, H**	
**1998**	**Sites 2 & 3**	**3**	**1843**	**9**	**R, EN, OF**	**T, H**	**D**[46], **C & M** [45]
1999		0	511	1			M [45]
2000	Site 1	0	391	0			M [45]
2001		0	731	2			
**2002**	**Sites 2 & 3**	**2**	**766**	**4**	**R, CSS**	**P, H, D**	
2003		0	330	1			
2004		1	389	1	R	H	
2005	Site 2	1	374	1	R	T	
2006		2	622	1	R, OF	T, C, H	
2007	Site 3	4	470	1	R, CSS	P, I, T, C	
**2008**	**Site 2**	**2**	**1032**	**4**	**R, CSS, OF, BG**	**H, E**	
2009		0	712	3	R, CSS, OF	T, H, E	
**2010**	**Sites 2 & 3**	**3**	**898**	**5**	**HT**	**H, E**	**D** [46]
2011	Site 3	6	396	0	R, CSS	T, H, E	
2012	Site 3	4	730	3	R, OF, CSS	H, E, S	D [45]
2013		1	379	0	OF, BG	H	

Community members identified years with severe flood events (see sites with severe flooding). Rainfall data (annual rainfall and days > 50 mm) are from the Granja Santa Ines weather station in Machala. Years with high total rainfall are bolded; they exceeded the upper quartile (740 mm/year) of total annual rainfall from 1990–2013. Disease outbreaks (dengue, malaria, cholera, typhoid) at the city level were identified from the Desinventar database [45] and previously analyzed Ministry of Health case data [46]. Flood events (n = number of events), causes, and impacts at the city level were extracted from the Desinventar database.

*Annual days of rainfall that exceeded 50 mm/day, the 99th percentile of daily rainfall during the rainy season (January-June, 1986–2015 baseline).

^1^Causes: rainfall = R, high tides = HT, El Niño = EN, canal or river overflow = OF, collapsed sewerage system = CSS, blocked drainage or garbage = BG

^2^Impacts: T = transportation interrupted, C = crops damaged, H = homes/property damaged, S = schools damaged, E = people evacuated, P = loss of power, I = infrastructure damage, D = human deaths, HH = health hazard (stagnant water)

^3^D = dengue fever, M = malaria, C = cholera, T = typhoid fever

**Fig 4:**
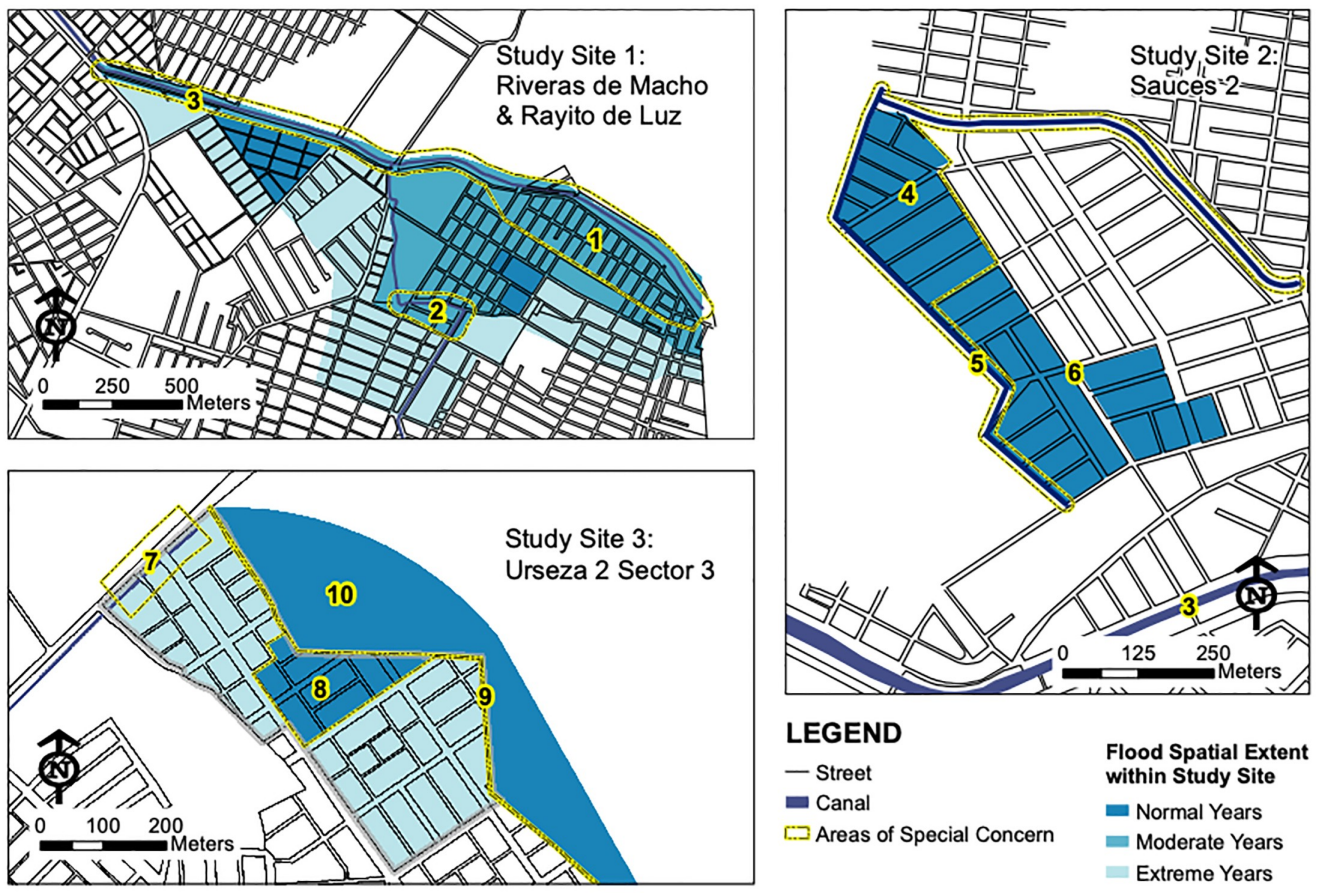
Flooding Extents within Study Areas. Maps generated by focus groups show the spatial extent of historic floods occurring within their communities. The ten areas of special concern included the following: (1) Places with strong currents during floods—these areas included police and fire stations and community health clinic; (2) inadequately-sized drainage pipes; (3) El Macho Canal—a tidal-influenced canal that acts as primary drainage canal for sewer and storm water systems and is a source of floodwater; (4) former shrimp farm—many parcels are unfilled and are full of water year-round; streets in this area have large persistent mud puddles that limit transit and pedestrians; (5) El Tigre Canal—a stagnant ditch that regularly overflows, flooding roads and private homes; (6) a primary road that remains dry during seasonal floods—this intersection also is used as a meeting area for community events; (7) inadequately-sized culverts; (8) a low-lying area that was formerly a brick quarry (known as “the hole”)—this site has an elementary school and private residences and endures annual flooding; (9) a berm constructed of uncapped material fill—material is being removed illegally and used as fill for private properties; and (10) an abandoned shrimp farm—stagnant pools collect water and periodically flood. This map was created using freely available street and neighborhood data from https://www.ecuadorencifras.gob.ec/geoportal/, and all other data generated in focus group conversations, rendered in ArcGIS, and image files created using Adobe software.

People identified and mapped the sectors of their neighborhood that flooded annually and during severe floods (Fig 4). This revealed spatial heterogeneity in perceived flood risk at the sub-neighborhood level. Each community mapped three to four areas of special concern such as places with strong currents during floods, canals that overflow, inadequate drainage systems, low lying areas with schools and homes, and other places that accumulate stagnant water (Fig 4). Participants at Site 3 identified a berm that was built recently to isolate floodwaters from the adjacent low-lying residential area. The berm was built without a concrete cap and individuals were removing fill material from the berm illegally for their personal use, thereby weakening the berm’s protective capacity. Participants were concerned that the berm would collapse during the next flood event, as described in the following:

“The government built this berm, but the people are not aware [of the purpose]: they remove the berm material to fill their own [properties]. They don’t know. The problem is that it endangers us all. Luckily it hasn’t rained hard recently”

*(Man from Site 3)*.

Community maps were compared to flood hazard maps generated by the SNGR. Most of the city area in the SNGR maps was classified at the highest hazard level including all of the study areas. High hazard was defined as: (1) low lying zones with slopes between 0–5% that remain flooded for more than 6 months during the year, and (2) areas where the accumulation of water is caused by rainfall as well as rising river levels during the rainy season. The spatial resolution of the SNGR maps did not allow for an analysis of differential flood hazard within the study sites.

Community members reported that proximity to blocked drainage areas was the most important geographic determinant of flood exposure. Official reports also identified blocked drainages and garbage as causes of flooding in 2008 and 2013, respectively [45] (Table 2). People explained that the blockages were caused by trash accumulation in areas that lacked regular municipal garbage collection and infrastructure (e.g. trash platforms). This resulted in a severe and perpetual litter problem when combined with the presence of stray dogs and illegal dumping at the city periphery. National census data confirmed that access to garbage collection was lowest in the urban periphery [48]; 63–83% of homes in the study had no garbage collection (Table 1). A participant described the combined effect of inadequate infrastructure, garbage, and flooding.

“On the street where I live, there’s a canal… That canal is now pure garbage, which clogs [the culvert] and stagnates it. This year we are afraid that it’s going to rupture… the pipe is very small”

*(Woman from Site 1)*.

Homes located near periurban estuarine canals were affected by tidal activity and overflowing canals, as were confirmed by official reports (Table 2). People explained that high tides impeded rainwater runoff during heavy rainfall events, resulting in flooding. Machala’s largest estuarine canal, “El Macho”, is one of the two main canals that transport the city’s runoff and untreated sewage into the ocean. Participants from Site 1 reported that as recently as 30 years ago, El Macho canal was a naturally flowing mangrove inlet, with clear water used for swimming, bathing and fishing. Increasing urban settlements in recent decades have augmented sedimentation and flow rates in the canal and led to the proliferation of formal and informal sewer drains flowing into the canal.

Community members identified low elevation as another key geographic factor that increased flood exposure. They stated that unfilled parcels of land collected standing water that could linger for months, or in some cases, year-round. Participants from Site 2 (Fig 4) identified year-round flood problems in properties that had not purchased material fill and lacked pumps and sewer systems to remove the pools of water.

“There is no [storm] sewer system…here it rains and it stagnates. It doesn’t have anywhere to go”

*(Woman from Site 2)*.

From 2002 onward, official reports frequently identified collapsed sewer systems as a cause of flooding. Community members explained that financial resources determined the ability of a family to raise the property’s elevation, as described in the following,

“Sometimes the [economic] situation of some families is so low, and there is no fill [because] there is no money”

*(Woman from Site 2)*.

Management of urban growth and development in low elevation flood plains was identified as a fundamental step to prevent flood exposure.

### Sensitivity to flooding

Participants perceived that certain demographic groups were more sensitive to flood-related health problems and economic/material losses. Children, the elderly, and the physically handicapped were identified as the most sensitive populations due to lower immunity to infections; decreased mobility; and reliance on others for medication, food, and water. Participants reported that children were exposed to contaminated floodwaters while playing outdoors. Low-income households were also identified as highly sensitive, since they were less likely to have a second floor of the home where they could seek refuge or store valuables during floods. They were also less likely to have a rooftop cistern with ample freshwater, and were less able to purchase fill to raise the level of their parcel. Residents who relied on public transportation systems were affected because they had to wade through floodwaters and mud during their daily commutes. Car owners also faced financial burdens due to damages to their automobiles. Other sensitive groups included people with animals (cats, dogs, poultry) and recent migrants who did not have nearby relatives to help them.

Flood sensitivity was not specifically recorded by any governmental dataset; however, we compared focus group results to the spatial distribution of a subset of sensitive demographic groups that were previously mapped using data from the most recent national census data (2010) [47,48]. Flood-sensitive groups (e.g. younger households, households in poor condition without access to piped water) were concentrated in the northern periphery of the city—the location of two of the three study sites. Certain sensitivity metrics identified by communities were not available in the census, such as household income, reliance on public transportation, and social isolation (individuals without nearby family/friends) (S2 Table).

### Impacts from flooding

People perceived that flooding presented a high social and economic burden on their community. Individuals reported that the primary impacts associated with flooding were damages to individual property (e.g., homes, cars, animals) and damages to people’s health (e.g., drowning, injury, and disease). Secondary impacts included the damages to roadways and destabilization of infrastructure and buildings. People’s livelihoods and daily activities were disrupted, and they struggled to pay for repairs to their homes. In official reports, damages to homes were the most frequently reported impact (13 of 17 years with flooding), followed by interruptions in transportation (8 years), evacuations (7 years), and crop damage (6 years) (Table 2). Government reports also noted power outages and damages to schools, but did not mention damages to personal vehicles or animals.

People perceived that exposure to flood water and mud and the resulting health impacts were a chronic problems. Specific health impacts included infectious diseases diseases, injury/drowning, and venomous snakebites. Mosquito-borne diseases, such as dengue fever, were mentioned as a risk factor for residents who lived in proximity to pools of standing water. Cholera and typhoid fever were also associated with flooding. Participants reported skin infections due to contact with flood and wastewater. Government data sources confirmed outbreaks of infectious diseases (cholera, typhoid fever, malaria, dengue fever) following heavy rainfall associated with El Niño events in 1992–1993, 1997–1998, and 2010 (Table 2). Of note, disease outbreaks were also documented in non-flood years, for example when the diseases were first emerging as new epidemics (e.g., dengue in 1990 and cholera in 1991). Thirteen deaths due to flooding were reported in 2002; however, causes were not specified. Additional information on drowning, injury, snakebites or skin infections was not available.

### Adaptive capacity across flood stages

Adaptive capacity actions identified by community members were compared to existing initiatives, as reported in focus groups (Table 3). In the response phase, the measures proposed by the community align with measures that were being implemented. Participants identified gaps in the recovery phase and prevention phase, as detailed in the following.

**Table 3:** Adaptive capacity actions before, during and after flood events.

	Actions identified by communities	Current interventions
**Prevention and preparation**
	Purchase fill to raise the elevation of low-lying properties	Elevation of roads and fill along the right of way paid for by municipal urban revitalization projects; road access measured by the census
	Stock up on canned food, bottled water and flashlights, and create flood emergency kits.	Educational initiatives by the SNGR
	Keep patios clean to prevent mosquito breeding and to maintain drainage	Educational initiatives by the Ministry of Health
	Community cleanups (“mingas”) to keep drainage canals clean	Ad hoc initiatives by neighborhood associations
	Regular trash pick-up to keep streets clean and drainage ways cleared	Trash collection is conducted by the municipal government. There are no formal educational initiatives; garbage collection access measured by the census
	Flood response simulations to lend support during times of emergency	Educational initiatives by the SNGR
	Listen to weather alerts through formal and informal media channels or via community sirens	SNGR information and alerts is transmitted by local news stations and radio channels
**Response**	Engage in evacuations	Evacuation efforts and flood abatement led by local, provincial, and national emergency responders in certain instances
	Create ad hoc drainage ditches	
	Gather sandbags	
**Recovery**	Employment to gather funds to rebuild/repair homes and other property	None identified; flood insurance programs do not exist
	Community, NGO, or governmental assistance to rebuild damaged homes	Ad hoc initiatives by neighborhood associations and Hogares de Cristo (NGO)

A comparison of actions identified by community members versus government interventions, as reported in focus groups.

During the flood preparation and prevention stage, participants identified limited economic resources (financial capital) and lack of community leadership (social capital) as underlying barriers to flood prevention. They also perceived that the community lacked the political voice and coordination (political capital) needed to obtain support for large-scale infrastructure improvements to reduce flood exposure, as expressed in the following:

There is no communication between authorities… People do not put pressure on them to do something good for the neighborhood. What people always want to do is to overthrow the government, not to put the pressure on them to do something good for the neighborhood”

*(Man from Site 3)*.

A leader commented on community fatigue and the difficulty in organizing people after a prolonged campaign to legally incorporate the neighborhood ended successfully, and residents became complacent,

“The moment that the mayor legalized the properties… nobody came to meetings. That is what happens… when it comes to a workshop, [now] no one has time. They will tell you, ‘I am busy.’… Now it takes a lot of effort to organize us”

*(Woman from Site 2)*.

Despite these challenges, participants from Site 3 had recently formed a neighborhood brigade that had been trained in flood response simulations by the SNGR, thereby increasing human capital. Most individuals, however, were unaware of this initiative, suggesting a lack of community engagement and the need for outreach and education. Another effective experience shared by community members was the recent tsunami EWS outreach campaign in Ecuador, where they received training and identified potential escape routes and meeting locations for their families in the case of a tsunami.

Participants discussed the effectiveness of the formal and informal flood warnings preceding the flood response stage. They indicated that formal communication outlets (e.g. television and radio) did provide general flood warnings based on forecasted rainfall as part of an existing official EWSF operated by the national government. However, they perceived that the forecasts were either too general or not timely enough. A participant described an ad-hoc warning system, based on upstream river observations,

“Sometimes, by chance, one travels from Guabo [a nearby town upstream from Machala] to see that the Jubones River is full. Then they will let us know that there is risk [of flooding] … It’s the only way [to know in advance], because the authorities do not warn us, and there are no alarms … nothing. The last time that the Jubones peaked and overflowed, [down] here was a scorching sun… It happens because the Jubones brings water from the mountains… and when the Jubones fills, and there is high tide here, we flood”

*(Woman from Site 3)*.

Other instances of informal flood warnings occurred through community communication channels on social media, such as Facebook, Twitter or WhatsApp. Participants proposed greater use of these social media to disseminate flood warning and response information. However, they cautioned that informal communication chains might not reach members of the community equally during times of crisis. Accordingly, all groups identified sirens or loudspeakers as the most effective way to alert people for flood evacuations.

With respect to the flood response stage, people perceived community and government actions to be relatively effective. Community members were mobilized during times of crisis to help their neighbors in need. However, enforcing mandated evacuations was difficult, according to focus groups, due to the real concern of looting and the lack of law enforcement. When asked how one decided to stay or go during a flood evacuation, one participant responded,

“You have to stay because if not, you will be left without anything… the thieves do not care. They jump into the water and that’s it”

*(Woman from Site 3)*.

During the flood recovery stage, community members struggled to take actions to repair the damages caused by flooding due to lack of financial capital. People said that they were responsible for bearing the cost of rebuilding their home, and they were unaware of flood insurance programs for private homeowners. Participants indicated that approximately one month of wages were lost during reconstruction efforts following severe flood events—a significant loss for a low-income family. Although they identified some programs that assist in rebuilding homes, these programs were highly competitive, middle class families were not eligible, and the construction was perceived as flimsy. The recovery stage was also limited by a lack of effective political engagement (political capital). They expressed distrust of local authorities whom they perceived to be more interested in political maneuvering than improving the welfare of the people.

“The authorities only come when there are votes and when it’s election time… They offer [funding] when there are elections… Or when there is a collapsed house or a drowning, but [even then] only that family is helped”

*(Man from Site 1)*.

Despite these challenges, they did describe moments of unity (social capital), for example when people at Site 1 came together to repair a neighbor’s home that had collapsed into the Macho Canal.

## Discussion

Coastal flooding incurs a high social and economic burden worldwide, and the impacts are projected to increase in urban areas [1,2]. Community perceptions can inform the implementation of tailored flood risk reduction strategies, such as those of the United Nations Sendai Framework for Disaster Risk Reduction to prepare, respond, and “build back better” [10]. Machala is an ideal case study for coastal flooding given the high hazard level, long history of flooding, community concern, available government data, and interest in improving EWSFs. The findings and the participatory approach applied here can inform practitioners and community members seeking to implement interventions to reduce flood exposure, particularly in other resource-limited urban areas around the world.

This study revealed persistent social-ecological vulnerabilities that increase the risk of flooding in the urban periphery, expanding on prior studies in the LAC region [9,16]. Low levels of adaptive capacity in LAC [51], particularly in urban areas [52] have been attributed to poor housing conditions, a lack of infrastructure, lack of decision-maker access to local data, and national policies that focus on mitigation rather than adaptation [11,19,53]. In this study, adaptive capacity was limited, in part, by a lack of social and political capital, and a lack of engagement by the government with community stakeholders. Studies show that social networks, familiar ties, and traditions may be less supportive and stable in urban areas as compared to rural communities [54,55]. Prior studies from Machala also found that marginalized communities in the urban periphery lacked legal standing and political access, which limited their ability to engage effectively with government institutions [56].

This study provides local insights into the escalating social injustices associated with development in low-lying coastal areas. With high development costs to elevate homes out of the floodplain, matched with land use policies that drive low-income communities into periurban low-lying areas, we hypothesize that the communities in this study bear a proportionately greater burden of flooding than wealthier communities. Maps of census indicators clearly show the geographic stratification of vulnerability across the urban landscape, as confirmed in prior studies in Machala [48].

To understand the gaps in adaptive capacity and flood risk reduction, the political context in Ecuador must also be considered. Within the last decade, the SNGR and the Secretary of Water (SENAGUA) were created during a period of relative prosperity and economic growth. However, political distance between national and local governments during this period hampered efforts to increase adaptive capacities at city and community levels. One mega project, the Pasaje-Machala flood control and water diversion project, is located in the El Oro province near the site of this study [57]. However, mega-hydraulic infrastructures were designed primarily to protect economic interests (banana crops and shrimp farming), with limited protection of citizens in cities. Between the low-lying city conditions, the inadequately designed drainage canals, limited governance during the extreme events, and the lack of coordination between the key actors, inter-institutional relations remain weak and have arguably weakened in recent years. In the context of these precarious institutional structures, this study reveals that (1) social cohesion and empowerment of community leaders is essential to increase local capacities to reduce flood risk, and (2) citizens have precise knowledge and contextual information that can be shared with government allies, such as city planners and risk managers, when an open and trusting relationship is established.

By triangulating focus group information with existing governmental data, we were able to identify instances when the datasets were complimentary, concordant, or discordant. The discrepancies in community flood reports—as compared to rainfall levels and official flood reports—may be attributed to localized flooding in specific low-lying locations or faulty memory. While memory driven data is predisposed to inaccuracies and over-simplicity, we found that the community-generated timelines did provide a useful conversation tool as well as a gauge for the relative impacts of episodic flooding. The official causes of flooding identified by the SNGR at the city-level were similar to the causes identified in communities; however, the focus groups additionally identified ten areas of special concern and provided local context. These areas of concern are clear policy targets for local decision makers—allowing for focused and impactful investment of limited risk management resources.

The community timeline of flooding spanned more years than the available meteorological or flood event data, and community flood maps were more detailed than SNGR flood hazard maps. Focus group data can supplement existing historic data on flooding, depending on the age distribution of focus group participants and their length of residency in the community. In areas with little historical data or lack of coverage by hydro-meteorological stations, the reconstruction of historic flood timeline and community mapping can serve as an information source for historic flood events, as well as to determine locations of high vulnerability. In instances where data are non-existent, focus groups may provide insights on periodic flooding cycles and localized factors that contribute to flooding. Indeed, this and other studies suggest that participatory methods can generate accurate quantitative data while capturing local priorities [58].

Regarding health outcomes, communities accurately perceived the role of flooding in triggering outbreaks of infectious diseases. They identified children as the most vulnerable group, and this is supported by recent epidemiological studies in Machala, which found that children under 10 bear the greatest burden of dengue illness [59]. Prior local field and modeling studies also support their perception that mosquito vectors and dengue transmission increase following heavy rainfall events, in particular those associated with El Niño [46,60]. However, their perception that dengue fever risk increased due to pools of stagnant floodwater is a misconception, as the mosquito vector inhabits receptacles with filled with rainwater or tap water around the home (e.g., buckets, used tires, rubbish in the patio); this misconception has been documented previously [56].

With respect to sensitive population groups, it is notable that recent migrants were identified as a high-risk group. Since conducting this study, 1.2 million refugees and migrants from Venezuela have passed through Ecuador and over 200,000 have settled in the country [61]. Many migrants have settled in Machala due to proximity to the southern border crossing at Peru. Many migrants lack social and financial capital and are unfamiliar with local climate hazards. In Machala, migrants are settling in precarious periurban neighborhoods, increasing the population at risk of flooding. As census data are updated once per decade, focus groups can provide more recent insights into local population dynamics and sensitivities that are not captured by the census, such as social isolation of recent migration populations.

### Opportunities and policy implications

Understanding local community perceptions is critical for policy makers interested in implementing flood risk reduction interventions. People’s perceptions of their risk or vulnerability influence their interest and ability to adopt risk reduction actions implemented by the government, as shown in prior studies of dengue fever [56]. Knowledge of the communities’ own risk perception is helpful for first responders to adequately communicate risk during times of disaster (e.g. emergency evacuations) or for officials who conduct outreach efforts to promote adaptive behaviors. Also, individuals who are aware of the impacts of flooding on their livelihood, assets, and health are more willing to take preventative measures rather than wait to take costly reactive measures [62]. Self-reported vulnerability may be indicative of a community’s motivation to self-initiate preparation actions for flooding events and adaptation to climate change [63]. The process of self-reporting further engages local communities in the process of education and raises awareness [39]. Policy makers should be concerned if communities have a low perception of the vulnerability relative to other communities in similarly vulnerable situations, as this turn amplifies overall social risk.

Local insights gathered in focus groups can assist in producing tailored solutions for a community, which in turn increases the feasibility and effectiveness of implementation actions. Focus groups can reveal less-obvious local issues that exacerbate flooding or render “expert-based” solutions ineffective. Without a corresponding effort to engage and gather data through integrated top-down and bottom- up approaches, interventions are unlikely to be sustainable [39,64].

This study has implications for the development of people-centered early warning systems. In 2006, the document “Developing Early Warning Systems: A Checklist” was developed to implement the early warning components of the World Conference on Disaster Reduction Hyogo Framework for Action 2005–2015: Building the Resilience of Nations and Communities to Disasters [65]. This document presents four key elements essential to people-centered early warning systems: 1) risk knowledge built upon systematic collection of data to address patterns and trends for a variety of hazards and vulnerability; 2) monitoring and warning services built upon accurate and timely scientific information; 3) clear and understandable dissemination and communication of risk information and early warnings to all members of the public; and 4) responses based on updated and tested plans that utilize local capacities and knowledge and are familiar to the public. The following findings and recommendations are elicited from this case study, and they link to the UN-ISDR Early Warning System checklist items.

1. Improve monitoring and accuracy of warnings. Flood warnings in Ecuador currently incorporate only rainfall data. An integrated monitoring network is needed, linking ocean and atmospheric data and forecasts. Recent studies suggest the potential to forecast El Niño events up to two years in advance [66], and these models are being used with an ensemble of climate forecasts to predict climate conditions and dengue fever epidemics in southern coastal Ecuador [67]. The incorporation of El Niño events and tidal data in flood hazard modeling for coastal areas is a critical component of management efforts along the Pacific coast of Latin America [9].
2. Develop clear flood warnings across a variety of communication channels. Our findings suggest that the use of both formal and informal communication networks may improve the delivery of EWSFs information. Flood forecasts issued via a system of sirens or loudspeakers, AM radio (used by taxi drivers) and individualized text alerts could complement existing television and radios channels. While sirens provide a warning to all individuals within hearing range, their effectiveness depends on whether community members know how to respond. Individualized text alerts could include detailed instructions on how to respond based on personalized information.
3. Leverage and strengthen existing community flood response capacities. An EWSF can leverage community resources by being thoroughly integrated in community-based initiatives and organizations. Community members indicated that they were capable of participating in work brigades, childcare, or training each other in preparation simulations. Education interventions should aim to increase local capacities, should be adequately staffed and resourced, and can explore the possibility of a train the trainer model, thereby increasing the capacity of local leaders.
4. Include principles of flood resiliency and risk prevention in comprehensive planning and land use regulations. Land-use zoning code and related land use policies should be enacted and enforced to dissuade residential development in hazardous areas like canal buffers and to use high-quality and appropriately-engineered flood management structures. Educational and collaborative planning opportunities exist at both local and municipal levels.

### Limitations

Since this qualitative case study focused on high-risk periurban areas, these findings cannot be generalized to communities that face a lower overall risk of flooding. Also, participants who opted to participate in this study may have been more motivated to do so based on their personal experience with flooding. Historical intensity and frequency of flooding are subject to the collective community memories. This case study was not used to prioritize a particular group by their vulnerability, but rather to present a nuanced, self-reported characterization of flooding in high-risk communities. In this way, the methodology provides key insights necessary to inform potential flood risk reduction actions.

## Conclusions

This study highlights the challenges and opportunities to reduce flood hazards in highly vulnerable coastal cities. The areas of special concern identified and mapped by community members are examples of clear local policy targets for flood risk reduction. The participatory approach presented here (1) provides important context to shape local policy and interventions in Ecuador, complimenting data gathered through standard flood reporting, (2) provides a voice for marginalized communities and a mechanism to raise local awareness, and (3) provides a research framework that can be adapted to other resource limited coastal communities at risk of flooding.

## Supporting information

S1 Table Codes for the analysis of focus group transcripts. Codes were developed based on vulnerability framework (Fig 2) and were used for qualitative analyses of transcripts.
(DOCX)

S2 Table Social vulnerability in Machala. This document compares the types of sensitive populations identified by focus groups to the data available from the national census.
(DOCX)
