# Supplementary material for: A participatory community case study of periurban coastal flood vulnerability in southern Ecuador
Source: PLoS One. 2019 Oct 25;14(10):e0224171. doi: 10.1371/journal.pone.0224171 (PMC6814235; doi:10.1371/journal.pone.0224171)
Supplement: S1 Table — Codes were developed based on vulnerability framework (Fig 2) and were used for qualitative analyses of transcripts. (DOCX) [file pone.0224171.s001.docx]

**S1 Table. Codes for the analysis of focus group transcripts.** Codes were developed based on vulnerability framework (Figure 2) and were used for qualitative analyses of transcripts.

| **Categories** |  | **Indicators** |
| --- | --- | --- |
| **Hazard** |  | Type of flood event |
|  |  | Time scale occurrence of flood event |
|  |  | Secondary hazards |
| **Exposure** |  | Proximity to hazard |
|  |  | Duration |
|  |  | Timing |
|  |  | Elevation |
|  |  | Land use |
| **Sensitivity** |  | Structural instability |
|  |  | Lack of access to dry areas |
|  |  | Low immunity to flood-related diseases |
|  |  | Lacking psychological/ physical abilities to cope/adapt to floods |
|  |  | Demographic groups with specific land-use needs |
| **Adaptive Capacity** | **Social Capital** | Effective multi-scale flood management structure |
|  |  | Public participation |
|  |  | Cohesion |
|  |  | Collective memory of previous floods |
|  |  | Surveillance |
|  |  | Social equity |
|  |  | Individual actions to prepare |
|  |  | Leadership |
|  | **Human Capital** | Knowledge of helpful flood actions |
|  |  | Training for flood response |
|  |  | Adequate # of individuals dedicated to flood management |
|  |  | System-wide accountability |
|  |  | Population density |
|  |  | Education |
|  |  | Literacy |
|  |  | Diversity of skills |
|  | **Physical Capital** | Equipment |
|  |  | Physical meeting space |
|  |  | Food and potable water access |
|  |  | Access to effective transportation |
|  |  | Communication infrastructure |
|  |  | Monitoring equipment |
|  |  | Potable water |
|  |  | Connection to public services |
|  |  | Housing/property type |
|  | **Financial Capital** | Sufficient budget resources available or dedicated for flood actions |
|  |  | Effectiveness in fund allocation |
|  |  | Insurance |
|  |  | Consistent Income/ Funding Source |
|  | **Natural Capital** | Functioning hydrologic systems |
|  |  | Secure pollution storage receptacles |
|  |  | Air quality |
|  |  | Weather/climate predictability |
