# Supplementary material for: A participatory community case study of periurban coastal flood vulnerability in southern Ecuador
Source: PLoS One. 2019 Oct 25;14(10):e0224171. doi: 10.1371/journal.pone.0224171 (PMC6814235; doi:10.1371/journal.pone.0224171)
Supplement: S2 Table — This document compares the types of sensitive populations identified by focus groups to the data available from the national census. (DOCX) [file pone.0224171.s002.docx]

**S2 Table. Social vulnerability in Machala.** Characteristics of sensitive populations identified by focus groups and corresponding data available from the national census [1].

| **Focus group: sensitive populations** | **Census data** |
| --- | --- |
| Children | Minors under the age of 15 |
| The elderly | Individuals aged 65 years or greater |
| Individuals with physical handicaps | Individual with physical handicaps |
| Low-income households | Not measured by Census |
| Households without access to water | Households without access to potable water |
| Households without access to sewer | Households without access to sewerage |
| Individuals reliant on public transportation | Not measured by Census |
| Car owners | Number of registered vehicles* |
| Individuals with livestock/pets | Type of occupation (agriculture, cattle raising |
| Social isolation (individuals without nearby relatives/friends) | Not measured by Census |

*Registered vehicles available at: https://www.ecuadorencifras.gob.ec/vehiculos-matriculados-serie-historica-2008-2014/

1. INEC. Censo de Poblacion y Viviendo [Internet]. Instituto Nacional de Estadistica y Censos (INEC); 2010. Available: http://www.ecuadorencifras.gob.ec/registro-de-descargas-cartograficas/
